# Incline dependence of the power-duration relationship in cross-country skiing

**DOI:** 10.3389/fphys.2025.1712475

**Published:** 2025-11-13

**Authors:** Marton Horvath, Erik P. Andersson, Adam Kölnas, Adam Spreitz, Hjalmar Boström, Arvid Sörfeldt, Dan Kuylenstierna

**Affiliations:** 1 Swedish Winter Sports Research Centre, Department of Health Sciences, Mid Sweden University, Östersund, Sweden; 2 Chalmers University of Technology, Gothenburg, Sweden; 3 Microwave Electronics Laboratory, Department of Microtechnology and Nanoscience, Chalmers University of Technology, Gothenburg, Sweden

**Keywords:** aerobic capacity, anaerobic capacity, critical power, performance prediction, performance testing, power output, sports performance, three-parameter critical power model

## Abstract

**Introduction:**

This study aimed to develop a methodology for establishing the power–duration relationship in cross-country skiers and to investigate the influence of incline on critical power ( CP ) model parameters.

**Methods:**

Twelve trained male cross-country skiers performed four constant work-rate predictive trials on a motor-driven treadmill, using the double poling sub-technique, to determine their power–duration relationships at 2° and 8° inclines in a randomized order. The testing protocol also included maximum speed tests performed at both inclines. Power-duration relationships were modeled using a modified expression of the three-parameter critical power model.

**Results:**

The derived power-duration relationships were significantly different between the two inclines. At an 8° incline, the estimated work capacity above CP (i.e., $W^{\prime }$ ) was more than two times higher than at a 2° incline ( 24.87±8.75 kJ vs. 7.07±1.61 kJ, respectively; Z=3.06 , P=0.002 , $r_{rb}=0.88$ ), which was partly explained by an increased anaerobic power capacity (i.e., $P_{an}$ = 4.82±0.64 W ⋅ kg^-1^ vs. 1.67±0.34 W ⋅ kg^-1^, respectively; Z=3.06 , P=0.002 , $r_{rb}=0.88$ ). Although CP estimates differed by approximately 16% between the two inclines on a group level ( 2.78±0.22 W ⋅ kg^-1^ vs. 2.39±0.74 W ⋅ kg^-1^ at a 2° and at an 8° incline, respectively), a moderate non-significant effect of incline was observed with large individual variances ( Z=1.88 , P=0.06 , $r_{rb}=0.54$ ). The incline had a non-significant effect on the time constant parameter estimates ( Z=1.57 , P=0.12 , $r_{rb}=0.45$ ), yet inter-individual variation remained considerable.

**Discussion:**

The findings demonstrate that in cross-country skiing, both $W^{\prime }$ and $P_{an}$ are highly incline-dependent, showing markedly higher values at steeper gradients. Moreover, the variability observed in CP and $W^{\prime }$ across inclines exceeded the typical sensitivity of these parameters to external factors reported in cycling. A large proportion of the incline-related changes in model parameters could be explained by accounting for the estimated variations in gross efficiency across speeds and inclines. However, the persistence of a significant difference in $W^{\prime }$ even when expressed in terms of estimated metabolic power at steeper inclines suggests the involvement of additional physiological mechanisms, potentially a larger amount of recruited muscle mass due to differences in muscle fiber recruitment between conditions.

## 1 Introduction

Although methods for estimating mechanical power output using positional data in cross-country skiing are well-established (for details, see, for example, Gløersen et al. (2018b); Swarén and Eriksson (2019)) and ski pole-integrated propulsive power output measurement is emerging (Johansson et al., 2019; Kuylenstierna et al., 2020), power output data inclusion into training and performance analysis remains limited in the sport. In contrast, cycling has widely adopted power output profiling over the past two decades, aided by commercial power meters, which have enabled accurate external training load assessment, power-based training prescription, and the continuous monitoring of changes in the athletes’ performance across racing seasons (Quod et al., 2010; Leo et al., 2022; Pinot and Grappe, 2010; Leo et al., 2021). Contrarily, training load monitoring in cross-country skiing is primarily based on the assessment of physiological and perceptual responses to exercise, such as heart rate, blood lactate accumulation, and the rate of perceived exertion (Seiler and Kjerland, 2006; Yu et al., 2023). Nevertheless, these measures have been shown to underestimate fatigue from short-duration anaerobic and neuromuscular efforts due to their time lag (e.g., sprints over short climbs, or position changes in a sprint race), as well as to struggle accurately capturing both the instantaneous magnitude of effort and fatigue accumulation during intermittent exercise (Bolger et al., 2015; Sitko et al., 2020).

Under competitive conditions, cross-country skiers frequently generate power outputs exceeding those associated with their maximal oxygen uptake, particularly during uphill sections, followed by flat or downhill segments that typically permit partial recovery (Gløersen et al., 2020; Andersson et al., 2017; Andersson et al., 2019). In addition to advancements in the available technology, this intermittent nature of cross-country ski races, characterized by both repetitive high-intensity bouts of effort taxing the anaerobic energy systems on climbs, and a continuously high demand for aerobic energy turnover along the race course (Losnegard, 2019), suggests that if accurate power output assessment were feasible, the critical power concept could be introduced into cross-country skiing as an alternative approach for investigating the energetic demands of the sport (Gløersen et al., 2020; Jones and Vanhatalo, 2017). However, given the concept’s physiological basis, the influence of external factors, such as incline and speed, on the ratio between external work rate and metabolic energy turnover (i.e., gross efficiency) must be considered when implementing it in cross-country skiing (Sidossis et al., 1992).

The relationship between sustainable power output and exercise duration is commonly characterized using critical power models, originally formulated by Monod and Scherrer (1965) to describe the hyperbolic dependence of work capacity on time to exhaustion. The two-parameter critical power model describes power output ( P ) as a function of exercise duration ( t ) as:

$$P\left(t\right)=CP+\frac{W^{\prime }}{t},$$ (1)

where CP is the so-called critical power, representing the boundary between the heavy- and severe-intensity domains, and $W^{\prime }$ is the finite work capacity above CP (Poole et al., 2016). To address the unrealistic prediction of close to infinite power output at near-zero durations, as follows from Equation 1, a negative time asymptote ( k ) was introduced into Equation 1 as a third parameter by Morton (1996), formulating the three-parameter critical power model as:

$$P\left(t\right)=CP+\frac{W^{\prime }}{t-k}.$$ (2)

The standard establishment of the power-duration relationship typically involves multiple constant work rate tests or time-based time trials, performed within the severe-intensity domain, which methodology results in the typical restriction of the accurately modeled exercise duration range to the 2–15 min domain (Bergstrom et al., 2014; Ferguson et al., 2010; Poole et al., 2016). Nevertheless, recent studies have demonstrated that using the three-parameter critical power model can extend the exercise duration range applicable to the model towards durations as short as 20 s (Vinetti et al., 2023; 2019). Furthermore, single-visit protocols have been developed and implemented for assessing CP and $W^{\prime }$ in cycling (Simpson and Kordi, 2017; Spragg et al., 2024).

The present study aimed to develop a methodological framework for establishing the power–duration relationship in cross-country skiers using a treadmill roller-skiing protocol. Furthermore, the study sought to examine how incline influences the derived parameter estimates of the used critical power model.

## 2 Materials and methods

### 2.1 Subjects

Twelve trained male cross-country skiers (mean ± SD: age 32 ± 6 years; body mass 77 ± 6 kg; height 1.82 ± 0.05 m; representing Tier 2 and 3 athletes according to McKay et al. (2021)) volunteered to participate in this study. To fulfil the inclusion criteria, all participants had to possess competition experience in either traditional or long-distance cross-country skiing and had to be familiar with treadmill roller skiing. Prior to their laboratory visit, the participants’ physical performance capacity was evaluated through a self-reported questionnaire. Before the testing procedure commenced, the subjects provided their written informed consent to participate. The study was preapproved by the Swedish Ethical Review Board (2023-03470-01) and was conducted according to the Declaration of Helsinki.

### 2.2 Equipment and testing procedure

The participants visited the laboratory on one occasion, during which they completed two incremental maximum speed tests and eight constant work rate predictive trials to exhaustion (a total of ten tests with target durations ranging from 5-320 s), using the double poling (DP) sub-technique. All tests were performed on a motorized treadmill (dimensions: 3.5 m × 2.5 m; Rodby Innovation AB, Vänge, Sweden) using the same pair of Swenor Alutech classic roller skis (length: 720 mm, mass: 2100 g/pair, wheel type: type 2; Swenor, Sarpsborg, Norway) mounted with Rottefella Xcelerator 2.0 bindings. The rolling friction coefficient ( $\mu_{r}$ ) was determined via a towing test following the methodology described by Sandbakk et al. (2010), measuring a rolling friction coefficient $\mu_{r}=0.018$ . The participants used ski boots of their choice and ski poles within 2.5 cm of their approved maximal length (FIS, 2016).

Each participant completed all tests at both 2° and 8° inclines in a randomized order. The protocol began with a 10-min standardized warm-up. After a 2-min recovery, a maximum speed test was initiated following the methodology described by Stöggl et al. (2007). At a 2° incline, the maximum speed test started at 16 km ⋅ h^-1^, while the treadmill speed was increased by 1 km ⋅ h^-1^ every 6 seconds after the initial stage. Meanwhile, at an 8° incline, the test commenced at 7 km ⋅ h^-1^, with 0.5 km ⋅ h^-1^ increments in a similar manner. The first stage of these tests lasted 20 s to account for the treadmill belt’s acceleration. The maximum speed tests were stopped by the test leader when the participant failed to maintain the treadmill speed (i.e., the roller ski front wheels dropped behind the midpoint of the treadmill). Maximum speed was defined as the speed at the last completed stage. After a 4-min rest, participants performed four predictive trials to exhaustion, with target durations of 5, 20, 80 and 320 s. Predictive trials with target durations of 5, 20, and 80 s were separated by 4 min of active/passive rest. Meanwhile, after the 80-s predictive trial, the participants rested for 12 min. After completing the predictive trial with a target duration of 320 s, a low-intensity cool-down and a 1-h recovery period followed. During this recovery period, the participants were offered 320 kcal of nutrition in the form of sports drinks and carbohydrate gels, and had the option to perform low-intensity exercise *ad libitum*. The same testing protocol was then repeated at the remaining incline, depending on the order of randomization.

The relatively slow treadmill acceleration (i.e., 0.5 km $\cdot {\text{h}}^{-1}\cdot {\text{s}}^{-1}$ ) posed a challenge during the short-duration predictive trials. To address this, the protocol was designed such that the participant held onto the front bar of the treadmill while it was accelerated to 80% of the target speed (see Section 2.4). Upon release of the bar, the test leader completed the acceleration to the required speed. Each trial began once the treadmill reached the target speed and concluded when the front wheels of the roller skis dropped behind the treadmill midpoint.

Body mass-normalized mean power output ( $\hat{P}$ ) during roller ski tests was calculated as the sum of power against gravity and friction as:

$$\hat{P}=g\left(\mu_{r}cos\left(\alpha \right)+sin\left(\alpha \right)\right)\cdot v,$$ (3)

where g is the gravitational acceleration (i.e., $9.81\;m/s^{2}$ ), α is the incline and v is the velocity of the treadmill.

### 2.3 The modified three-parameter critical model

Morton introduced an abstract negative time-asymptote into the two-parameter critical power model to create a y-intercept for the power curve, thereby estimating maximal instantenous power output. If τ=−k , Equation 2 can be expressed as:

$$P\left(t\right)=CP+\frac{W^{\prime }}{t+\tau },$$ (4)

where τ is the time constant governing how quickly power output declines toward CP , thus mainly affecting the curvature of the power curve. Nevertheless, this model (i.e., Equation 4) lacks uniformity in the dimension of considered metrics due to its simultaneous consideration of both work and power; a problem that can be resolved by defining a new parameter - the anaerobic power capacity ( $P_{an}$ ) - as follows:

$$P_{an}=\frac{W^{\prime }}{\tau }.$$ (5)

When introducing Equation 5 into Equation 4, it can be reformulated as:

$$P\left(t\right)=CP+\frac{P_{an}}{1+t/\tau },$$ (6)

standardizing the dimensions of the critical power model parameters. This reparametrization dismisses the negative time constant from the model, enhancing its physiological interpretability. Equation 6 can be further reformulated by defining the ratio $r=P_{an}/CP$ , resulting in:

$$P\left(t\right)=CP\left(1+\frac{r}{1+t/\tau }\right).$$ (7)

**FIGURE 1:**
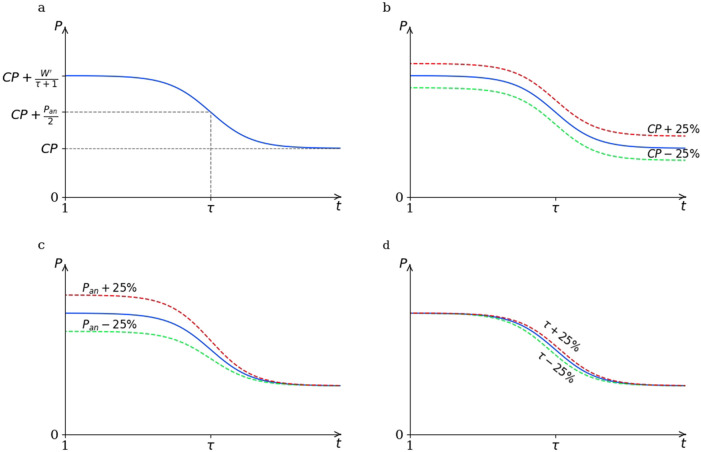
Panel **(a)** Representation of model parameters, including the components of $W^{\prime }$ (i.e., $P_{an}$ and τ ), of the used critical power model with a logarithmic time scale on the horizontal axis. Panels **(b–d)** An illustrated effect of ± 25% variation in the critical power ( CP ), the anaerobic power capacity ( $P_{an}$ ) and the time constant ( τ ) model parameters on an arbitrary power-duration curve.

The sigmoid shape of the log-transformed power-duration curve is governed by the specific model parameters in distinct ways (Figures 1a–d). A change in CP results in the vertical shift of the entire curve, thereby modifying power output values across all exercise durations (Figure 1b). In contrast, the parameter $P_{an}$ primarily affects power output values at shorter durations (i.e., t<τ ) (Figure 1c). Meanwhile, τ influences the power output values around the curve’s inflection point (i.e., governing the curvature of the hyperbolic power-duration curve), having a pronounced effect near τ but minimal impact on values far from this point (Figure 1d). These model characteristics were considered in the design of target durations for predictive trials. To obtain model parameter estimates, Equation 6 was fitted to the power-duration data set from the predictive trials using least-square approximation in MATLAB (The MathWorks, Inc., Natick, MA, United States).

### 2.4 Predictive trials

The constant work rate predictive trials for assessing individual power-duration relationships aimed to induce complete exhaustion around specified target durations of 5, 20, 80 and 320 s, aligning with the characteristics of the used three-parameter critical power model (see Section 2.3). Due to the instrumental constraints posed to treadmill roller-skiing, the power output for each predictive trial had to be estimated in advance of testing. This estimation was based on the power output attained during the final stage of the maximum speed tests, calculated using Equation 3. Based on Equation 7, assuming that power output at level N equals $P_{N}$ , the power output at level N+1 was expressed using the following recurrence relation:

$$P_{N+1}=P_{N}-\frac{r\tau \left(t_{N+1}-t_{N}\right)}{\left(t_{N}+\tau \left(1+r\right)\right)\left(t_{N+1}+\tau \right)}P_{N},$$ (8)

where $t_{N}$ and $t_{N+1}$ are the respective target durations of predictive trials (for derivation see Supplementary Material). Before prescribing work rates for levels N≥2 , model parameters r and τ as well as the required work rate at the shortest test duration (i.e., $P_{1}$ ) had to be determined. Since there was no knowledge available on the magnitude of these parameters before the tests, the initial assumptions of these values (i.e., r=0.52 and τ=50 sec for a 2° incline and r=1.55 and τ=50 sec for an 8° incline) for the first participants were set utilizing data from cycling (Coggan, 2006; Quod et al., 2010) and publicly accessible data on ski ergometry exercise (Concept2, 2024). As the testing proceeded, the initially assumed parameters were dynamically adjusted to the average values of all participants who had completed the test earlier, which made the model effectively “self-trained”. The attained maximum speed was utilised to determine the required power output for $P_{1}$ (and, therefore, for all the subsequent levels). Based on the authors’ practical insights and preliminary testing, it was established that at a 2° incline, the treadmill speed corresponding to $P_{1}$ should be 5% higher than the power output corresponding to the attained maximum speed. Meanwhile, at an 8° incline, this speed was raised to 120% of this power output.

### 2.5 Gross efficiency adjustment concerning speed and incline

Gross efficiency (GE)−power  output relationships were derived concerning incline and speed using previously collected and published data by Andersson et al. (2017), assuming linearity for data points representing the four highest speeds and inclines. This process yielded the following linear regression equations:

GE(v)=−0.0654P(v)+31.041, (9)

for describing the influence of treadmill speed, and

GE(α)=−0.0048P(α)+18.989 (10)

for describing the influence of treadmill incline on GE . These relationships were used to predict GE values corresponding to mean power outputs during the predictive trials at both 2° and 8° inclines, enabling the subsequent estimation of metabolic power ( MP ) in the N th predictive trial as follows (Andersson et al., 2017):

$$MP_{N}=\frac{P_{N}}{GE_{N}}.$$ (11)

### 2.6 Statistical analysis

Due to the relatively small sample size ( n ), the Wilcoxon signed-rank test was used to detect differences in power output, time-to-exhaustion, and critical power model parameters across inclines (Blair and Higgins, 1985). To quantify the effect of incline, rank-biserial correlation coefficients ( $r_{rb}=Z/\sqrt{n}$ ) were calculated (Tomczak and Tomczak, 2014). Bland–Altman plots were used to assess the mean difference ± 95% limits of agreement between model parameters (Bland and Altman, 1999), while Pearson correlation coefficients ( r ) were used to evaluate relationships between model parameters and power output across inclines. Variance in the model parameters across inclines was assessed using stepwise multiple linear regression, and model performance was quantified by the adjusted coefficient of determination ( $R_{\text{adj}}^{2}$ ). To describe changes in model parameters across inclines in detail, the participants were ordered and divided into high and low performers based on their summed maximum speeds and additional comparisons were performed. The power-duration curve's fit was evaluated using the coefficient of determination ( $R^{2}$ ), and parameter precision was expressed as the standard error of estimate ( SEE ) and coefficient of variation ( CV% ) according to Black et al. (2017). The precision in model parameter estimates (i.e., CP , $P_{an}$ and τ ) was compared between inclines using the Wilcoxon signed-rank test. Additionally, root mean square error ( RMSE ) was used to quantify model accuracy. Statistical analyses were conducted in SPSS v29.0 (IBM Corp., Armonk, NY, United States). Data are presented as mean  ±  SD. Significance level was set to α≤0.05 .

## 3 Results

In the final stage of the maximum speed tests, the participants reached 27.4  ±  2.0 km ⋅ h^-1^ at a 2° incline and 12.7  ±  1.1 km ⋅ h^-1^ at an 8° incline, indicating mean test durations of 88.5±11.9 and 88.0±13.4 seconds, respectively ( Z=−0.28 , P=0.78 , $r_{rb}=0.08$ ). Corresponding mean power outputs were 4.01  ±  0.21 W ⋅ kg^-1^ and 5.49  ±  0.44 W ⋅ kg^-1^, respectively, resulting in a significant difference ( Z=3.01 , P=0.002 , $r_{rb}=0.88$ ) and a strong correlation across inclines ( r=0.87 , P<0.001 ). Consequently, power output was significantly higher at an 8° incline compared to a 2° incline during all predictive trials ( Z=3.06 , P=0.002 , $r_{rb}=0.88$ ; Table 1). However, incline manipulation did not significantly affect time-to-exhaustion between 2° and 8° trials ( P>0.05 for all).

**TABLE 1:** Power output and time-to-exhaustion during predictive trials ( $P_{1}$ - $P_{4}$ ). The required work rates were prescribed based on the attained maximum speed in the maximum speed tests using Equation 8.

Incline	Power output [W ⋅ kg^-1^]		Time-to-exhaustion [s]	
	2°	8°	2°	8°
$P_{1}$	4.19 ± 0.30	6.62 ± 0.57^*^	9.6 ± 2.7	8.9 ± 2.3
$P_{2}$	3.94 ± 0.29	5.80 ± 0.51^*^	23.8 ± 3.5	26.2 ± 6.7
$P_{3}$	3.44 ± 0.25	4.46 ± 0.45^*^	91.9 ± 28.6	96.2 ± 46.9
$P_{4}$	3.06 ± 0.22	3.41 ± 0.37^*^	405.8 ± 263.3	358.6 ± 266.8

$P_{1}$ , $P_{2}$ , $P_{3}$ , and $P_{4}$ represent predictive trials with target durations of 5, 20, 80, and 320 s, respectively. ^*^ significantly different values compared to a 2° incline ( P≤0.01 ).

**FIGURE 2:**
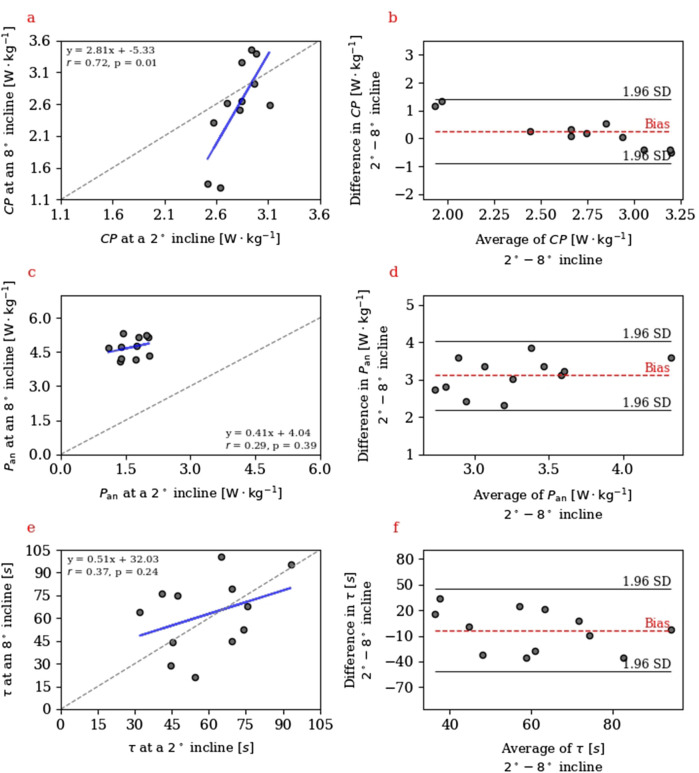
Panel **(a,c,e)** Linear regressions between critical power ( CP ), anaerobic power capacity ( $P_{an}$ ) and time constant ( τ ) model parameters at a 2° and an 8° incline. Panel **(b,d,f)** Bland-Altman plots representing the bias and 95% Limits of Agreement of model parameters (i.e., CP , $P_{an}$ and τ ) at the investigated inclines.

$P_{an}$ was almost two times higher at an 8° incline compared to a 2° incline ( 4.82±0.64 W ⋅ kg^-1^ vs. 1.67±0.34 W ⋅ kg^-1^, Z=3.06 , P=0.002 , $r_{rb}=0.88$ ). In contrast, CP tended to be higher at the 2° incline relative to the 8° incline ( 2.78±0.22 W ⋅ kg^-1^ vs. 2.39±0.74 W ⋅ kg^-1^), although this difference did not reach statistical significance ( Z=1.88 , P=0.06 , $r_{rb}=0.54$ ). Furthermore, the incline had a non-significant effect on τ , with higher values observed at an 8° incline ( 65.5±17.3 s vs. 56.1±13.6 s, Z=1.57 , P=0.12 , $r_{rb}=0.45$ ). Bland-Altman analyses revealed relatively large limits of agreement for all model parameters between incline settings, especially concerning CP and τ . The mean difference (i.e., bias) for CP , $P_{an}$ and τ were 0.26±0.58 W ⋅ kg^-1^ (−0.88–1.41 W ⋅ kg^-1^), 3.11±0.47 W ⋅ kg^-1^ (2.19–4.04 W ⋅ kg^-1^) and −3.2±24.8 s (−51.7 to 45.3 s), respectively (Figure 2). Furthermore, the demonstrated changes in model parameters $P_{an}$ and τ implied that the elicited $W^{\prime }$ values of 7.07±1.61 kJ at a 2° incline increased significantly at an 8° incline up to 24.87±8.75 kJ ( Z=3.06 , P=0.002 , $r_{rb}=0.88$ ).

The expansion of $P_{an}$ (i.e., $\Delta P_{an}$ ) alone accounted for approximately 31% of this variance ( $\Delta W^{\prime }\;\left[J\cdot {kg}^{-1}\right]=112.73\Delta P_{an}\;\left[W\cdot {kg}^{-1}\right]-127.21,\;R_{\mathrm{adj}}^{2}=0.31,\;P=0.035$ ), whereas the difference in τ (i.e., Δτ ) across inclines explained 70% of the observed variation in $W^{\prime }$ ( $\Delta W^{\prime }\;\left[J\cdot {kg}^{-1}\right]=3.93\Delta \tau \;\left[s\right]+189.77,\;R_{\mathrm{adj}}^{2}=0.70,\;P<0.001$ ). When $\Delta W^{\prime }$ was modeled as a function of both $\Delta P_{an}$ and Δτ , the explained variance increased further to 86% ( $\Delta W^{\prime }\;\left[J\cdot {kg}^{-1}\right]=76.75\Delta P_{an}\;\left[W\cdot {kg}^{-1}\right]+3.43\Delta \tau \;\left[s\right]-46.47,\;R_{\mathrm{adj}}^{2}=0.86,\;P<0.001$ ). No statistically significant linear relationship was found between $\Delta P_{an}$ and Δτ ( r=0.26 , p=0.41 ). When dividing the sample based on the summed maximum speed into high- and low-performing sub-groups ( $42.3\pm 1.8\;km\cdot h^{-1}$ vs. $37.9\pm 1.9\;km\cdot h^{-1}$ , respectively), it was revealed that CP was significantly different between inclines for the low-performing sub-group ( Z=1.99 , P=0.046 , $r_{rb}=0.57$ ), whereas $P_{an}$ was significantly different for both sub-groups ( Z=2.20 , P=0.03 , $r_{rb}=0.64$ for both), and τ showed no difference between inclines in any of the sub-groups ( P>0.05 for both; Table 2).

**TABLE 2:** Model parameters extracted from power-duration relationships at 2° and 8° incline.

Incline	CP [W ⋅ kg^-1^]		$P_{an}$ [W ⋅ kg^-1^]		τ [s]	
	2°	8°	2°	8°	2°	8°
All participants^a^	2.78 ± 0.22	2.39 ± 0.74	1.67 ± 0.34	4.82 ± 0.64^*^	56.1 ± 13.6	65.5 ± 17.3
High performers^b^	2.90 ± 0.18	2.70 ± 0.74	1.82 ± 0.25	4.96 ± 0.42^*^	52.4 ± 15.6	64.4 ± 14.0
Low performers^c^	2.66 ± 0.2	2.08 ± 0.66^*^	1.53 ± 0.38	4.68 ± 0.83^*^	59.9 ± 11.4	66.7 ± 21.4

CP , critical power; $P_{an}$ , anaerobic power capacity; τ , time constant.

^a^ all participants ( n=12 ).

^b^ first six participants based on their summed maximum speed across inclines (i.e., $42.3\pm 1.8\;km\cdot h^{-1}$ ).

^c^ last six participants based on their summed maximum speed across inclines (i.e., $37.9\pm 1.9\;km\cdot h^{-1}$ ).

^*^significantly different values compared to a 2° incline ( P<0.05 ).

Predicted GE values for 2° and 8° inclines in the predictive trials with target durations of 5, 20, 80 and 320 s were 9.9±1.5% vs. 16.5±0.2% , 11.2±1.5% vs. 16.9±0.2% , 13.7±1.3% vs. 17.3±0.2% , and 15.6±1.1% vs. 17.7±0.1% , respectively (*GE* values were predicted based on Equations 9, 10). Across the predictive trials predicted GE increased by 36±6% at a 2° incline, while it rose by 7±1% at an 8° incline. Fitting Equation 6 to the metabolic power values estimated based on Equation 11 decreased the difference in derived $W^{\prime }$ values between the two inclines from ∼250% to ∼64% compared to mechanical power output (i.e., $W^{\prime }=68.1\pm 16.6$ kJ vs. 111.5±49.7 kJ in terms of metabolic power at 2° and 8° inclines, respectively). On the other hand, the proportional difference in CP between inclines was nearly identical regardless of whether mechanical power output or metabolic power was considered (i.e., a 0.7% difference in mean values), demonstrating approximately 10% higher values at the 2° incline, but not a statistically significant difference ( P>0.05 for both; Figure 4).

Concerning the accuracy and precision of the derived power-duration relationships, the extracted model parameters resulted in an excellent fit ( $R^{2}=0.996\pm 0.005$ ) and accurate prediction of prescribed power outputs at both inclines ( RMSE=0.053±0.048 W ⋅ kg^-1^). On the other hand, the precision in critical power model parameter estimates was significantly lower at an 8° incline compared to a 2° incline ( $CP:\;CV{\%}_{2{}^{\circ}}=3.1\pm 1.4$ vs. $CV{\%}_{8{}^{\circ}}=9.7\pm 4.4$ , Z=2.67 , P=0.01 , $r_{rb}=0.78$ ; $P_{an}:\;CV{\%}_{2{}^{\circ}}=4.9\pm 6.7$ , vs. $CV{\%}_{8{}^{\circ}}=6.7\pm 4.0$ , Z=0.65 , P=0.52 , $r_{rb}=0.19$ ; and $\tau :\;CV{\%}_{2{}^{\circ}}=26.3\pm 13.6$ vs. $CV{\%}_{8{}^{\circ}}=30.6\pm 20.3$ , Z=0.42 , P=0.68 , $r_{rb}=0.12$ ).

**FIGURE 3:**
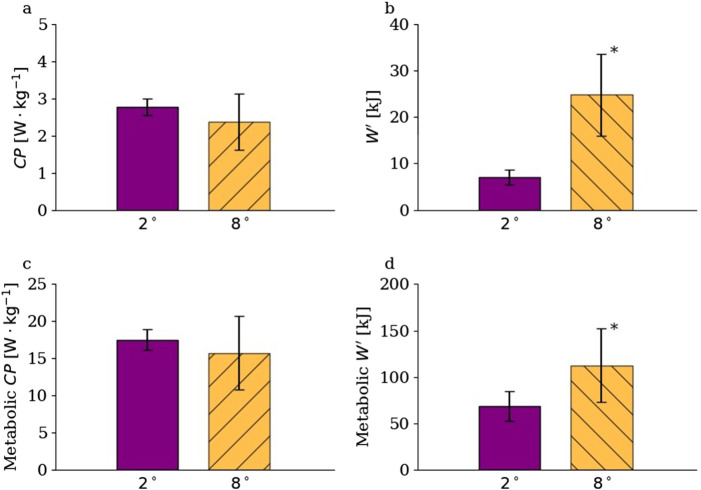
Critical power ( CP ) and the work capacity above critical power ( $W^{\prime }$ ) in terms of mechanical power output and mechanical work [panel **(a,b)**], as well as metabolic power and metabolic work [panel **(c,d)**] across inclines. ^*^ marks significantly different values ( P<0.01 ).

## 4 Discussion

This study is the first to apply the critical power concept to performance assessment and prediction in cross-country skiing. The study introduced a novel testing approach based on the three-parameter critical power model, and aimed to investigate the effect of incline on critical power model parameters during double poling on a motorized treadmill. Its main findings were that: 1) $W^{\prime }$ and $P_{an}$ showed a substantial increase with a steeper incline, whereas 2) CP demonstrated a moderate decrease at an 8° incline, although this decrease was not statistically significant. Accounting for the estimated effect of changes in GE across predictive trial conditions lowered the difference in $W^{\prime }$ between inclines, but it remained statistically significant.

**FIGURE 4:**
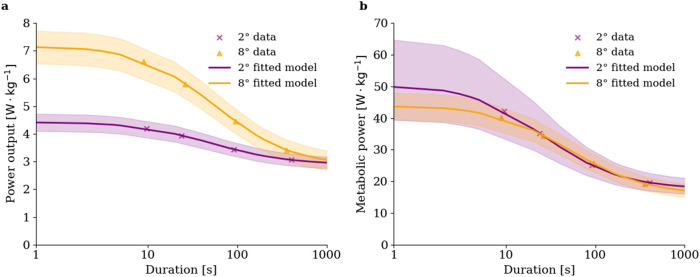
Power-duration [panel **(a)**] and estimated metabolic power-duration [panel **(b)**] relationships at 2° and 8° inclines. Metabolic power was estimated using power output-gross efficiency relationships derived from data published by Andersson et al. (2017). Shaded areas represent power-duration relationships fitted against mean power output values ±SD .

In the current study, the participants demonstrated a considerably higher (i.e., ∼40% ) power output in the maximum speed test at an 8° incline compared to a 2° incline, which implied the prediction of consistently higher power outputs for predictive trials at the former incline, aiming to elicit exhaustion over the corresponding target durations (see Sections 2.2, 2.4). In terms of the derived critical power model parameters, this increase in power output was reflected by a substantial increase in both $P_{an}$ and $W^{\prime }$ , whereas a moderate but non-significant decrease of CP was also found as the gradient rose (Figure 4). This suggests the notable implication that the power-duration relationship is incline dependent in cross-country skiing. In contrast, previous research investigating the effect of incline on critical power model parameters in cycling has found a relatively small but significant difference in $W^{\prime }$ but no effect on CP across incline conditions (Hovorka et al., 2022).

The conversion of power output into metabolic power closed the distance between data points representing predictive trials in the power-duration plane by accounting for the estimated effect of changing GE across speed-incline conditions (Figure 4). Using metabolic power instead of power output in modeling the power-duration relationship resulted in a considerably decreased difference in metabolic $W^{\prime }$ estimates between inclines, showing that the estimated change in GE was one, but likely not the only physiological reason behind the observed incline-dependency, as $W^{\prime }$ was still significantly different across inclines (Figure 3). As $W^{\prime }$ has a fundamental anaerobic component (Puchowicz et al., 2018), a potential expansion of the anaerobic energy turnover could also play a role in the observed increase in this parameter. Previous research in treadmill roller skiing and running has shown that during maximal intensity exercise, an increase in gradient, and thus in mechanical load, results in a greater accumulated O_2_-deficit until the complete utilization of the anaerobic capacity is reached (Olesen, 1992; Karlsson et al., 2018). This effect can be partly attributed to the increased recruitment of muscle fibers due to the increased mechanical resistance (Sloniger et al., 1997); further supported by Bangsbo et al. (1993), highlighting the central role of active muscle mass in anaerobic energy turnover. In contrast, Wakeling et al. (2006) demonstrated that motor unit recruitment during locomotion can be adjusted to meet the mechanical demands of contraction, supported by the findings of Haase et al. (2024), who reported that during 10-s isokinetic cycling sprints the rate of blood lactate accumulation increased at higher cadences. Such a phenomenon may indicate an altered muscle fiber recruitment pattern at near-maximal skiing speeds on low inclines—where poling time has been shown to approach 200 ms (Stöggl and Holmberg, 2016) — resulting in a relatively greater proportional contribution of type II muscle fibers to the total power output.

It is important to highlight that our findings showed greater variability in CP than those reported in cycling by Hovorka et al. (2022), though the derived CP values ( ∼3.2−4.2 W ⋅ kg^-1^) align with data from similarly trained cyclists at both inclines (Chorley et al., 2020; Rossi et al., 2023), considering the generally ∼ 30%–40% lower GE in double poling compared to cycling (Millet et al., 2002; Andersson et al., 2021; 2017). Concerning external power output, the magnitude of $W^{\prime }$ was considerably smaller at a 2° incline (i.e., ∼ 7 kJ) compared to values reported in cycling, but values at an 8° incline were more representative of those or even slightly higher (i.e., ∼ 25 kJ) than values previously reported for cyclists at similar performance levels (Chidnok et al., 2012; Caen et al., 2024). Dividing the sample into two subgroups revealed differences in how incline affected CP . High performers showed only a slight decline in CP with increased incline, while low performers exhibited a more marked reduction. However, the declined performance observed in low performers may also be attributed to a potentially lower level of muscular strength and/or technical adaptations compared to high performers, leading to decreased muscular efficiency and GE at an 8° incline (Barrett-O’Keefe et al., 2012).

Altogether, the power–duration relationship carries implications that are more relevant to cross-country skiing than cycling, namely, the incline dependence of critical power model parameters, especially $W^{\prime }$ and $P_{an}$ . From a technical perspective, this might be explained by the fact that in cycling, GE is generally maintained across inclines and speeds due to gear shifting (however, it is influenced by power output and cadence), which allows riders to adjust crank torque, and maintain their optimal cadence irrespective of terrain conditions (Millet et al., 2002; Ansley and Cangley, 2009; Ettema and Lorås, 2009; Barker et al., 2006).

## 5 Methodological considerations

The present study has exclusively focused on the DP sub-technique. The selected testing gradients were 2° and 8°. Although skiers typically employ the diagonal stride sub-technique at the steeper incline due to its higher GE (Andersson et al., 2021; Løkkeborg and Ettema, 2020; Ettema et al., 2018), the choice of inclines was motivated by the known importance of upper-body power and strength concerning both sprint and distance skiing performance across sub-techniques (Staib et al., 2000; Østerås et al., 2016; Øfsteng et al., 2018). Furthermore, in long-distance cross-country ski races, such as the Visma Ski Classics (i.e., typically ranging from 50 to 90 km), skiers have adopted the exclusive use of the DP sub-technique—even on the steepest climbs of these race courses (i.e., representing maximum inclinations >8° ) — which enables the use of skis without grip wax and thereby improves overall performance (Stöggl et al., 2018).

Unlike conventional approaches prescribing predictive trial intensities relative to power output corresponding to maximal oxygen uptake (Bergstrom et al., 2014; Ferguson et al., 2010; Vanhatalo et al., 2007), predictive trial intensities in the present study were derived using the average power output attained during the final stage of the maximum speed tests and the modified three-parameter critical power model (see Section 2.3). Furthermore, the longest predictive trial was designed to remain well within the severe-intensity domain. This approach reduced total testing time to minimize fatigue accumulation, though it led to the inclusion of predictive trial durations representing the extreme-intensity domain. Concerning the chosen mathematical model, the findings of Vinetti et al. (2019) suggested that the three-parameter critical power model provides a valid approach for modeling power output across the chosen target durations, which has been applied previously to fit power data in the extreme-intensity domain (Vinetti et al., 2023). Nonetheless, based on previous findings, the formulation and implementation of a ‘multi-domain’ critical power model may offer improved predictions across the exercise intensity spectrum (Alexander et al., 2019; Hill et al., 2002; Puchowicz et al., 2020; Puchowicz and Skiba, 2025).

Time-to-exhaustion during the predictive trials was not significantly different between inclines, which suggests the reliability of the developed method for assessing the power-duration relationship across inclines during treadmill roller skiing. However, the mean deviation from the target durations remained substantial ( 24.3±14.1% ). The participants’ data suggest that this discrepancy could be reduced by fine-tuning the parameters of Equation 8, setting r=0.53 and τ=50.6 s for the 2° predictions, and r=1.55 and τ=56.6 s concerning power output prediction at an 8° incline, while increasing the prescribed power outputs to 108% and 126% of the power associated with the attained maximum speed for the 5-s predictive trials, respectively. The precision of model parameter estimates (i.e., CP , and $W^{\prime }$ ) appeared reasonable when compared to previously published data (Black et al., 2017), considering that three-parameter critical power models are expected to inflate the SEE . On the other hand, direct comparisons of parameter estimate precision between the two- and modified three-parameter models are not feasible concerning either CP or $W^{\prime }$ . Notably, the estimated τ parameters exhibited substantially lower relative precision than CP and $P_{an}$ , which can be explained by the nonlinear nature of Equation 6 concerning τ and the relatively low number of predictive trials around τ duration.

Derived model parameter estimates, especially CP , should be interpreted with caution due to the lack of fatigue assessment throughout the predictive trials. There is a potential that fatigue has contributed to the observed significant difference in CP in the low-performing subjects, but not in high-performing subjects, potentially due to their better aerobic fitness/fatigability. Another notable limitation of the present metabolic power estimates lies in the potential inter-individual variability in gross efficiency ( GE ). Although GE –power output relationships derived from an independent sample may provide realistic approximations of relative differences between experimental conditions, individual deviations are expected due to differences in athletes’ morphological, physiological, technical, and training characteristics. Despite these limitations, the estimations were essential for contextualizing the influence of incline on the establishment of the power–duration relationship. An effect particularly relevant in sports such as cross-country skiing, where the conversion of metabolic to mechanical power output is strongly influenced by environmental and biomechanical factors.

## 6 Practical applications

The limited availability of power meters for cross-country skiing and the potential inaccuracies related to positional data-based power output estimation limit the practical applicability of the present study, as its findings are primarily relevant to treadmill roller skiing. Nevertheless, the presented methodology has direct applications for performance testing in a laboratory environment. Monitoring changes in cross-country skiers’ power-duration relationship could provide more detailed insights into their individual strengths and weaknesses. Furthermore, exploring the dependence of the power–duration relationship on external factors, such as incline, could enhance performance analysis and provide new approaches for optimizing performance and pacing strategies in the sport.

## 7 Conclusion

This study demonstrated that incline during treadmill roller skiing has a substantial effect on the estimates of critical power model parameters, particularly $W^{\prime }$ and $P_{an}$ , which increased more than twofold at an 8° incline compared with a 2° incline.

Furthermore, the findings highlight the importance of accounting for external factors, such as incline and speed, when assessing the exercise-induced metabolic demand at a given mechanical power output in the context of establishing the power–duration relationship. These effects were shown to be particularly relevant in cross-country skiing, but may also have implications for cycling. Future studies should investigate how the accumulated $O_{2}$ deficit and other physiological parameters, including oxygen uptake, the depletion rate of muscular energy substrates, and the accumulation of fatigue-related metabolites, are affected by variations in speed, incline, and sub-technique selection during cross-country skiing, as well as how these factors influence critical power model parameters, since such analyses were beyond the scope of the present study.

## Data Availability

The raw data supporting the conclusions of this article will be made available by the authors, without undue reservation.
