# Supplementary material for: Incline dependence of the power-duration relationship in cross-country skiing
Source: Front Physiol. 2025 Nov 13;16:1712475. doi: 10.3389/fphys.2025.1712475 (PMC12657177; doi:10.3389/fphys.2025.1712475)
Supplement: Supplementary file 1 [file DataSheet1.pdf]

frontiers

Supplementary Material

1 DERIVATION OF THE PRESENTED RECURRENCE RELATION

As a first step in deriving the presented recurrence relation (i.e., Equation 8), Equation (7) should be rewritten as:

$$P(t) = CP\,\frac{t + \tau(1 + r)}{t + \tau}. \tag{S1}$$

Hence, the difference in maximal sustainable power output between two subsequent levels (i.e., N and $N + 1$) can be formulated as:

$$P_{N+1} - P_N = CP\left(\frac{r}{1 + \frac{t_{N+1}}{\tau}} - \frac{r}{1 + \frac{t_N}{\tau}}\right). \tag{S2}$$

Factoring out r and rewriting the expression on the right-hand side using a common denominator in Equation (S2) results in:

$$P_{N+1} - P_N = rCP\,\frac{\frac{t_N - t_{N+1}}{\tau}}{\left(1 + \frac{t_N}{\tau}\right)\left(1 + \frac{t_{N+1}}{\tau}\right)}. \tag{S3}$$

After multiplying the fraction's numerator and denominator on the right-hand side in Equation (S3) by τ, P_{N+1} can be expressed as:

$$P_{N+1} = P_N - rCP\,\frac{t_{N+1} - t_N}{\left(t_N + \tau\right)\left(t_{N+1} + \tau\right)}, \tag{S4}$$

where P_N can be expressed based on Equation (S1) resulting in:

$$P_{N+1} = P_N - \frac{r\tau\left(t_{N+1} - t_N\right)}{\left(t_N + \tau(1 + r)\right)\left(t_{N+1} + \tau\right)}\,P_N, \tag{S5}$$

as presented in Equation (8).
